# Demonstration of Resolution of Community-Acquired Pneumonia Over a Short Course of Antibiotics on [ ^18^ F]FDG-PET/CT Undertaken for Suspected Perimyocarditis Evaluation

**DOI:** 10.1055/s-0044-1788074

**Published:** 2024-07-04

**Authors:** Parth Baberwal, Sunita N. Sonavane, Sandip Basu

**Affiliations:** 1 Radiation Medicine Centre, Bhabha Atomic Research Centre, Mumbai, Maharashtra, India; 2 Homi Bhabha National Institute, Mumbai, Maharashtra, India

**Keywords:** community-acquired pneumonia, [ ^18^ F]FDG-PET/CT, antibiotics, perimyocarditis, early treatment response

## Abstract

A 28-year-old male presenting with left-sided pleuritic chest pain, cough, palpitation, and fever with mild ST depression in II, III, and aVF, raised troponin I, troponin T, creatine phosphokinase-MB, and erythrocyte sedimentation rate was referred for F-18 2-fluoro 2-deoxyglucose positron emission tomography with noncontrast computed tomography ([ ^18^ F]FDG-PET/CT) to rule out perimyocarditis. The first scan revealed incidental finding of [ ^18^ F]FDG avid left lobar pneumonia and inadequate myocardial suppression, thus perimyocarditis could not be ruled out. The clinician was informed and after counseling, patient consented for a repeat study post-high fat-low carbohydrate diet. A regional [ ^18^ F]FDG-PET/CT on the 5th day revealed adequate myocardial suppression, ruling out perimyocarditis. However, the metabolic and anatomical resolution of previously noted left lobar pneumonia was also observed in such a span of time with the administered antibiotics.

## Introduction

Pneumonia is the inflammation of one or both lungs' parenchyma, most commonly secondary to infections. The causative agents may be bacteria, viruses, fungi, and parasites. Clinically, they may present with cough (the most common), fever, tachycardia, pleuritic chest pain, and dyspnea on exertion, etc. 1 Evaluation is done by clinical assessment, chest X-ray, computed tomography (CT) chest (in complicated cases), and bronchoalveolar lavage. Management is conservative and includes antibiotics, hydration, and nutrition. F-18 2-fluoro 2-deoxyglucose ([ ^18^ F]FDG) is the most commonly used positron emission tomography (PET) tracer worldwide, mainly for oncological purposes, though strictly not limited to it given its nonspecificity and its established role in infection, cardiology, and neurology. 2 We herein present a case of suspected perimyocarditis with left-sided chest pain, normal electrocardiogram (ECG), and two-dimensional echocardiography (2D ECHO) and a raised troponin I value, who was incidentally detected to have pneumonia on [ ^18^ F]FDG-PET/CT done for perimyocarditis evaluation and had complete resolution within 5 days on a repeat study.

## Case Report

**Fig. 1:**
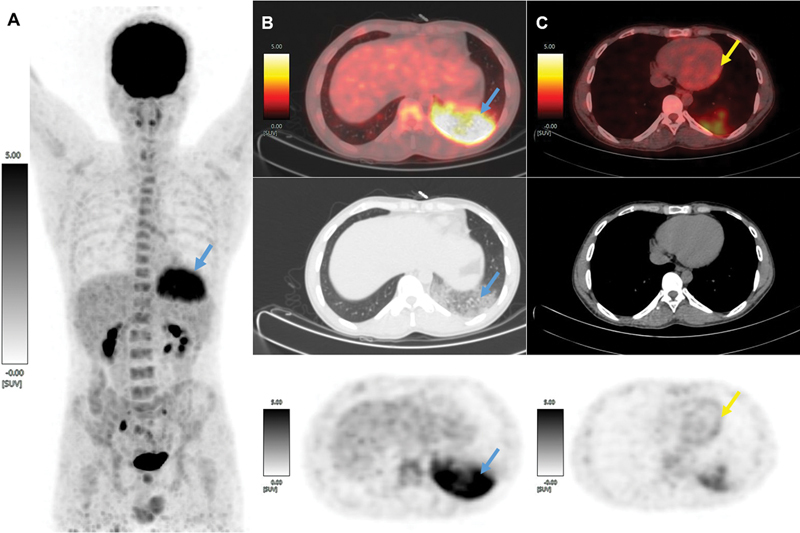
Maximum intensity projection of F-18 2-fluoro 2-deoxyglucose positron emission tomography with noncontrast computed tomography ([ ^18^ F]FDG-PET) scan of a case of suspected perimyocarditis with prolonged fasting and intravenous low molecular heparin loading 15 minutes prior to [ ^18^ F]FDG injection revealed abnormal [ ^18^ F]FDG uptake in the lower lobe of the left lung (marked with blue arrow of maximum standardized uptake value [SUVmax] 8.18) ( **A** ). A column of images ( **B** ) showing axial view of fused [ ^18^ F]FDG-PET/CT, CT only (showing diffuse ground-glass opacities in the lower lobe of the left lung), and [ ^18^ F]FDG-PET only, respectively (top to bottom), demonstrating avid uptake in left lower lobe, which suggests an infective etiology (marked with blue arrow). A column of images ( **C** ) showing physiological [ ^18^ F]FDG uptake in myocardium on axial views of fused [ ^18^ F]FDG-PET/CT, CT only, and [ ^18^ F]FDG-PET only images, respectively (top to bottom), suggesting improper preparation for evaluation of perimyocarditis (marked with yellow arrow).

**Fig. 2:**
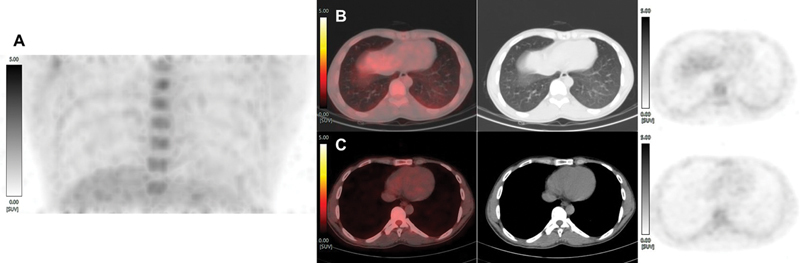
Maximum intensity projection of a repeat F-18 2-fluoro 2-deoxyglucose positron emission tomography with noncontrast computed tomography ([ ^18^ F]FDG-PET) scan (regional) ( **A** ) after following 48 hours of strict high fat-low carbohydrate diet and low-molecular weight heparin injection 15 minutes prior tracer injection showing complete resolution of increased [ ^18^ F]FDG uptake in the lower lobe of the left lung. Row of images ( **B** ) of axial view of fused [ ^18^ F]FDG-PET/CT, CT only, and [ ^18^ F]FDG-PET only (from left to right) showing a complete metabolic and anatomical resolution of metabolically active ground-glass opacities in the lower lobe of the left lung over a short course of antibiotics (5 days). A note can be made of complete myocardial suppression of FDG uptake in the row of images ( **C** ) of axial view of fused [ ^18^ F]FDG-PET/CT, CT only, and [ ^18^ F]FDG-PET only (from left to right), ruling out perimyocarditis.

A 25-year-old Indian gentleman, with no comorbidities and no addictions, presented to hospital with fever associated with cough with expectoration, chills and rigors, palpitation, headache, burning micturition, and sharp left-sided chest pain for 1 day. Vitals of the patient were stable. ECG showed sinus tachycardia with mild ST depression in lead II, III, and aVF. 2D ECHO showed a left ventricular ejection fraction of 60% with no other significant changes. The chest roentgenogram did not show any abnormalities. Blood workup showed hemoglobin (15 g/dL), total leukocyte count (14,800 cells/mm ^3^ ), C-reactive protein (100.72 mg/L), creatine phosphokinase-MB (283 IU/L), troponin T spot test was positive, and troponin I (9996.9 ng/L) were raised. Urine routine, urine culture, and blood culture were unremarkable. The patient was referred for [ ^18^ F]FDG-PET/CT to rule out perimyocarditis. The patient underwent only prolonged fasting of 12 hours followed by unfractionated heparin loading by an intravenous route (50 IU/kg) 15 minutes prior [ ^18^ F]FDG injection. The acquisition of [ ^18^ F]FDG-PET/CT was done 1 hour later. The scan showed [ ^18^ F]FDG avid (maximum standardized uptake value 8.18) ground-glass density in the lower lobe of the left lung ( Fig. 1 ), suggestive of lobar pneumonia with inadequate myocardial suppression as indicated by a low-grade [ ^18^ F]FDG uptake throughout the myocardium. The clinician was informed prior regarding the finding of pneumonia and inadequate myocardial suppression; further with consent, the patient was counseled for high fat-low carbohydrate diet (HFLC diet) for a minimum of 48 hours for evaluation of myocardium, postfatty meal preparation and repeat [ ^18^ F]FDG-PET/CT was acquired at 5th day from first scan. In the follow-up [ ^18^ F]FDG-PET/CT ( Fig. 2 ), complete myocardial suppression was noted, thus ruling out perimyocarditis; however, additionally, the previously noted [ ^18^ F]FDG avid ground-glass density in the lower lobe of the left lung was resolved, metabolically and anatomically. On discussion with the physician, history of intravenous antibiotics, viz., injection ceftriaxone 1 g twice daily and injection levofloxacin 500 mg twice daily, was obtained, one dose of which administered 1 day prior the first [ ^18^ F]FDG-PET/CT scan. [ ^18^ F]FDG-PET and CT clearly revealed no residual infection and with confirmed imaging documentation, the clinically recovered patient could be discharged. The patient was asymptomatic and doing well at the time of writing of this report.

## Discussion

The present case report demonstrates the antibiotic response of community-acquired pneumonia over a short course of antibiotics on [ ^18^ F]FDG-PET/CT. Evolution of pneumonia can be broken down into four phases: congestion, red hepatization, gray hepatization, and resolution. 3 Stage of resolution and restoration of the pulmonary architecture start by the eighth day in its natural course. Achievement of resolution phase is accelerated by antibiotics, which is well-visualized in our report showing anatomical and functional response of the infection within 4 days of initiation of antibiotics. CT component, along with [ ^18^ F]FDG uptake and clinical findings also helped in achieving some confidence about the etiology of infection being bacterial and initiation of prompt antibiotics. 4 5 [ ^18^ F]FDG is taken up by GLUT-1 receptors located on the surface of activated granulocytes, lymphocytes, and monocytes. During inflammation, cytokines are released and they upregulate GLUT-1 on activated inflammatory cells. 6 This enhances the uptake of [ ^18^ F]FDG during infection and inflammation, therefore can aid in assessment of sarcoidosis, tuberculosis, occult infection, autoimmune fibrosis, pneumonia, cystic fibrosis, and acute respiratory distress syndrome. 7

Another thing that can be emphasized in this case is the importance of preparation prior to [ ^18^ F]FDG-PET imaging in cases where cardiac inflammation/infection is suspected. Scans with diffuse myocardial uptake are usually considered nondiagnostic, as physiological tracer uptake of myocardium may mask any pathological tracer concentration. To reduce such physiological cardiac [ ^18^ F]FDG uptake, particular preparations like prolonged fasting, HFLC diet, and unfractionated heparin injection prior [ ^18^ F]FDG-PET imaging have been used. 8 9 The heart preferentially uses free fatty acids and lactate for energy production, while glycolysis produces only 30% of the substrates of the Krebs cycle. 10 Under the prolonged fasting condition, free-fatty acid mobilization from adipose tissue increases while glucose production and glucose oxidation decrease. 11 Unfractionated heparin activates lipoprotein lipase, which in turn increases free-fatty acid levels in the blood. 12 Therefore, all of the above methods help in suppressing physiological myocardial [ ^18^ F]FDG-PET uptake, which can be used conjunctively, as was employed effectively in our case for the second [ ^18^ F]FDG-PET study.

## Conclusion

We report a case with incidental finding of pneumonia on [ ^18^ F]FDG-PET/CT and spontaneous anatomical and metabolic resolution of left lobar pneumonia in repeat [ ^18^ F]FDG-PET/CT done following proper preparation to achieve proper myocardial suppression to rule out perimyocarditis.
